# Wettability Changes Due to Nanomaterials and Alkali—A Proposed Formulation for EOR

**DOI:** 10.3390/nano11092351

**Published:** 2021-09-10

**Authors:** Samhar Saleh, Elisabeth Neubauer, Ante Borovina, Rafael E. Hincapie, Torsten Clemens, Daniel Ness

**Affiliations:** 1 Montanuniversität Leoben, DPE Department Petroleum Engineering, Franz-Josef-Straße 18, 8700 Leoben, Austria; samhar@outlook.de; 2 OMV Exploration & Production GmbH, OMV Upstream Technology & Innovation, TECH Center & Lab, 1020 Vienna, Austria; Elisabeth.Neubauer@omv.com (E.N.); Ante.Borovina@omv.com (A.B.); Torsten.Clemens@omv.com (T.C.); 3 Evonik Operations GmbH, Research, Development & Innovation, D-63450 Hanau, Germany; daniel.ness@evonik.com

**Keywords:** EOR, nanoparticles, alkali, spontaneous imbibition, wettability, IFT, phase behavior

## Abstract

We investigated the usage of two silica nanomaterials (surface-modified) and alkali in enhanced oil recovery through Amott spontaneous imbibition tests, interfacial tension (IFT) measurements, and phase behavior. We evaluated the wettability alteration induced by the synergy between nanomaterials and alkali. Moreover, numerical analysis of the results was carried out using inverse Bond number and capillary diffusion coefficient. Evaluations included the use of Berea and Keuper outcrop material, crude oil with different total acid numbers (TAN), and Na_2_CO_3_ as alkaline agent. Data showed that nanomaterials can reduce the IFT, with surface charge playing an important role in this process. In synergy with alkali, the use of nanomaterials led to low-stable IFT values. This effect was also seen in the phase behavior tests, where brine/oil systems with lower IFT exhibited better emulsification. Nanomaterials’ contribution to the phase behavior was mainly the stabilization of the emulsion middle phase. The influence of TAN number on the IFT and phase behavior was prominent especially when combined with alkali. Amott spontaneous imbibition resulted in additional oil recovery ranging from 4% to 50% above the baseline, which was confirmed by inverse Bond number analysis. High recoveries were achieved using alkali and nanomaterials; these values were attributed to wettability alteration that accelerated the imbibition kinetics as seen in capillary diffusion coefficient analysis.

## 1. Introduction

Nanoparticles (NPs) have drawn the attention of the oil industry after proven success in many other fields of science, with enhanced oil recovery (EOR) as one of the crucial upstream applications. Research has mainly focused on silica nanoparticles (SiNPs) in EOR applications [1,2,3,4,5,6,7,8], due to their cost efficiency and environmental friendliness, making them a first choice. Bare SiNPs alone are not effective as EOR agents due to their negatively charged surface [1]. With adequate surface modification, SiNPs can penetrate and interact with the reservoir system, leading to a mobilization of the trapped oil and hence an increase in ultimate recovery. This increase could be driven by several mechanisms, among others: (1) a reduction in interfacial tension (IFT) between oil and brine [2,3,5], (2) the emulsification of the oil and stabilization of the formed emulsions [9,10], (3) pore channel plugging [11], (4) wettability alteration [2,5,8,10,12], or a combination of these. Nanoparticles (NPs) are unlike other chemical EOR (cEOR) agents, less liable to degradation under reservoir conditions [13]. In addition, NPs can act in synergy with other cEOR agents, allowing them to improve their effects like foam stabilization [14] or rheology enhancement [15]. 

Sharma et al. [16] observed that IFT was reduced in a brine/oil system by almost half of the initial, after adding 1 wt.% of SiNPs. Ahmed et al. [17] reported that IFT in an oil/brine system decreased by three times its original value after surface-treated SiNPs were added to the brine. They observed that IFT reduction was more significant when the concentration of NPs was increased. Neubauer et al. [9] showed that more negatively charged silica NPs were more effective in terms of IFT reduction than more neutrally charged silica, reporting a strong dependency on the nanoparticle’s particle size. Particles with a smaller size have a larger specific area and the particles have higher diffusivity due to the electric repulsion and Brownian motion [18]. By reducing the interfacial tension in the oil/brine system, emulsification can occur. The formed emulsion can act as a flow barrier to divert the flow into unswept zones and block the highly permeable corridors. Emulsion-based mobility control can be an adequate alternative to polymers, especially for heavy oil reservoirs [19]. The emulsification process can be achieved using surfactant, alkaline or both. The formed emulsion (e.g., surfactant-based) is liable to degradation under harsh reservoir conditions and that degradation can be in the form of coalescence, Oswald ripening, sedimentation, or creaming [20].

Binks and Rodrigues [21] showed that synergistic stabilization of oil-in-water (O/W) emulsions can be achieved by mixing oppositely charged NPs and surfactant. Kamkar et al. [22] investigated the rheology of oil-in-water emulsion formed by combining surface-modified NPs and hydrolyzed polyacrylamide (HPAM) polymer. Their results revealed that partially hydrophobic silica NPs have more favorable mobility from the bulk phase towards the oil–water interfaces, making them more effective in lowering IFT. 

Wettability alteration is a complex synergistic process that could be the outcome of different mechanisms. Polymers, for instance, can alter the wettability by a reptation mechanism, which leads to detachment of trapped oil droplets [23]. Chengara et al. [24] studied the utilization of NPs to detach an oil drop from a solid surface. They suggested that the nanofluid forms a wedge-shaped thin film due to the existence of disjoining pressure. It is worth mentioning that wettability alteration due to the spreading of nanofluid is a well-observed phenomenon, but the mechanism behind it still remains a research topic that has not yet been clarified. Disjoining pressure requires high concentrations of nanoparticles at the interface [25] and the nanoparticles should not be adsorbed on the rock surface [26]. Moreover, a water-wet rock surface is a prerequisite for the three-phase contact region [27]. Hence, structural disjoining pressure cannot solely serve as an explanation for wetting behavior and recovery mechanism using nanofluids.

NPs can alter the properties of rock mineral surface towards superhydrophilic (affinity for water) or superhydrophobic (repel water) based on how their surface was coated. Hydrophilicity promotes a water-wet surface [28], while hydrophobic NPs have almost no impact on the wettability [29]. Alvarez-Berrios et al. [30] investigated the effect of surface charge of silica NPs on the wettability alteration using Berea sandstone outcrops. The authors found through contact angle measurements that slightly negatively charged SiNPs reduced the contact angle from oil-wet to water-wet (106.3° to 74°). Berea sandstone in contact with negatively and positively charged NPs remained neutrally wet (94.5° and 100.2°, respectively). In combination with spontaneous imbibition experiments (wettability alteration), they reported that the highest ultimate recovery was achieved using low concentrations of slightly negatively charged silica NPs. However, when highly negatively charged NPs or positively charged NPs were used, higher concentrations were more effective in terms of wettability alteration. 

Bila et al. [31] performed core flood experiments using nanofluids containing polymer-coated silica NPs at low concentrations on neutrally wet Berea core plugs followed by an Amott-Harvey test (wettability). The authors showed that the highest additional recovery can be achieved by using NPs in secondary mode. They attributed the findings to various mechanisms, namely interfacial tension (IFT) reduction, the formation of micro emulsions and, most importantly, wettability alteration.

Another element of importance is the possible adsorption of the nanoparticles in the porous media, which have been studied also in relation to wettability. Nanoparticle adsorption is controlled by many factors. Li et al. [32] observed by the mean of advanced surface wetting visualization, an increase in NP adsorption onto Berea sandstone when the NP concentration rose. The pH of the nanofluids also had an impact on the adsorption process, with low pH (pH = 2.01) favoring uniform NP adsorption. As the nanofluids’ pH increased to 4.84, a thicker layer of NPs adsorbed onto the rock. Xu et al. [33] reported an improvement in the oil recovery and imbibition rate (Amott imbibition) after introducing silica NPs to the utilized brine. The oil recovery showed a dependency on the NP concentration, with the incremental recovery linked to IFT reduction and a lower contact angle, which promoted more water wetness. Wang et al. [34] studied the imbibition of fluids with silica NPs into a capillary using molecular dynamic simulation. The authors concluded that the displacement of nanofluids was higher with increased hydrophilicity.

Alkali has been used for many years as the EOR agent. Alkaline agents like NaOH or Na_2_CO_3_ are injected alone or as pre-flush for surfactants and polymer [35,36]. When injected as pre-flush slug, alkali reduces the polymer adsorption. Sheng [36,37] describes the mechanics of alkali for EOR, as well as in combination with other cEOR agents. Similar mechanisms taking place during alkali flooding occur in nano EOR, which mostly depends on the rock/oil/brine system. In previous work, we evaluated the nanomaterials alongside with alkali (Na_2_CO_3_) and with polyacrylamide polymers as emulsion stabilizers [9,10]. This included the evaluation of possible effects of other nanoparticles, alkali, and polymer [9,10] or alkali polymer [38,39] on wettability, IFT, and phase behavior.

There is a growing body of literature [11,40,41,42,43,44,45,46] endorsing the need to utilize a combination of EOR methods to obtain further benefits, which would translate into additional oil being recovered. We focus here on the synergistic effects of nanomaterials and alkali as a novel EOR formulation. Hence, Table 1 shows a literature overview related to the EOR methods discussed here. To the best of our knowledge, the synergistic effects of nanomaterials and alkali have not been investigated in detail. In this work, we compiled a representative comparison to explain the possible relation between spontaneous imbibition and interfacial tension when applying nanoparticles and alkali. With this study we aim to bridge knowledge gaps on the impact of surface-modified silica nanomaterials on enhanced oil recovery with a special focus on wettability alteration, by conducting interfacial tension measurements, phase behavior tests, and spontaneous imbibition tests. 

The paper is organized into five main sections: (1) the overall approach is presented; (2) the description of materials and methods; (3) the general approach of the paper; (4) the presentation of laboratory and modeling result; and (5) a summary and conclusions of the findings are presented.

## 2. General Paper Approach

To evaluate the effects of nanoparticles combined with alkali on wettability changes and IFT, we followed a series of steps that are described below and serve as a structure for understanding the results:

1. Fluid/rock selection and characterization: As a continuation of the work previously performed by some of the authors [9,10,38], we have selected two surface-modified silica nanomaterials (characterized by means of ζ potential) and Na_2_CO_3_ alkali, two brines (differing in ion content), and two oils (differing in TAN). Outcrop rocks (differing in clay content) were selected/characterized by routine core analysis (RCA);
2. Interfacial tension (IFT) measurements: IFT was measured as a function of time at 60 °C using a spinning drop tensiometer to define fluid–fluid interactions. Multiple concentrations and combinations were tested;
3. Phase behavior experiments: These supported an understanding of the emulsion volumes generated by the fluid–fluid interactions. This work is complemented by the data presented by Neubauer et al. [9];
4. Amott spontaneous imbibition experiments: Performed to evaluate wettability alteration and oil recovered over time by the imbibed fluid (nanoparticles/alkali). Data was assessed through detailed tracking of produced oil with time and final oil recovery;
5. Modeling of selected data: Data was modelled by means of the capillary diffusion coefficient and the inverse Bond number to link the findings to literature data.

## 3. Materials and Methods

### 3.1. Fluids and Rock Material

#### 3.1.1. Synthetic Brines

To investigate the effect of divalent cations, two synthetic brines were used, hereafter called softened injection brine (TW) and synthetic formation brine (FW). A detailed composition of each brine can be found in Table 2.

#### 3.1.2. Crude Oil

Crude oil samples from the 16 TH reservoir of the Matzen field (Well Bo-112) and the St. Ulrich reservoir (Well StU 65) were used in this study. As goal we investigate the influence of the total acid number (TAN) on the recovery process. The 16 TH oil has a more acidic nature than St. Ulrich oil. The 16 TH oil is also richer in the polar compounds (asphaltene and resins) than St. Ulrich oil, which is expected to promote more oil wetness. The properties of crude oil samples are listed in Table 3. 

#### 3.1.3. Nanomaterials 

**Figure 1:**
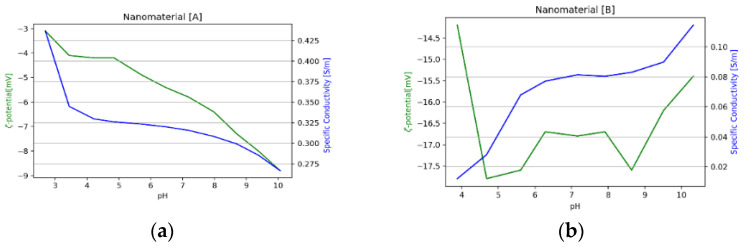
ζ potential of both nanomaterials with the respective specific conductivity; type A shown in (**a**) and type B in (**b**). ζ potential varies with the pH value of the solution. As demonstrated in (**a**), ζ potential of type A varies slightly within the pH range compared to type B. Type B is more negatively charged and its ζ potential changes within the same pH range as seen in figure (**b**).

**Figure 2:**
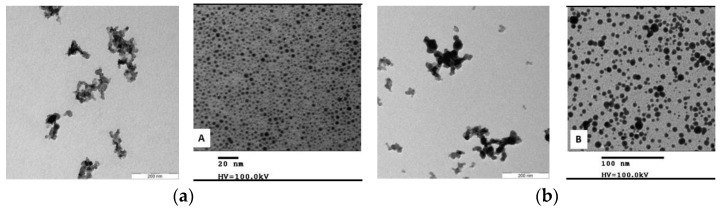
Transmission electron microscopy (TEM): nanomaterial type A shown in (**a**) and nanomaterial type B in (**b**).

Nanomaterials A and B were provided by Evonik Operations GmbH (Hanau, Germany) in form of a dispersion. They exhibit a size of about 110 nm (see Table 4), and their concentration in distilled water varies from 22.5 to 27.9 wt.%. The base material of both is fumed amorphous silica (AEROSIL^®^ fumed silica, Evonik Hanau - Germany). The surface of nanomaterial A is coated with polyethylene glycol (PEG), while the surface of nanomaterial B has a diol functionality. ζ potential was assessed using a Nano Z manufactured by Malvern Zetasizer Pro (Malvern Panalytical Ltd., Malvern - United Kingdom), which relies on using the technique of dynamic light scattering (DLS). The dispersion was titrated over a pH range (3–10) as shown in Figure 1. The PEG-coated nanomaterial type A has a zeta potential of between −3 and −8.5 mV. The diol-coated nanomaterial type B exhibits a zeta potential which is lower (−14 to −18 mV) and rather constant over the whole pH range (Figure 1). With a pH of around 10, that is typical for alkaline flooding according to French and Burchfield [49]; the zeta potential is ~−8 mV (nanomaterial type A), and −16 mV (nanomaterial type B). Figure 2. shows Transmission Electron Microscopy (TEM) (Jeol 2010F, 200 KV, Tokyo Japan) images of the nanomaterials. The structure of the fumed silica with high fractal dimensions is clearly visible. 

#### 3.1.4. Nanomaterials Combined with Alkali—Combined Solutions 

Different fluid combinations were prepared. Alkaline solutions were prepared with a concentration of 3000 ppm of Na_2_CO_3_, which was the concentration with maximum emulsion being created with 16 TH oil [9,10]. In addition, Lüftenegger and Clemens [50] found that using 16 TH oil, any higher alkali concentration would not result in further reduction of interfacial tension when used together with other chemicals. Based on previous evaluations [9], it was found that solutions with 0.1 wt.% of nanomaterials were optimum for stabilizing emulsions created due to alkali. Therefore, all experiments in this study were performed with 0.1 wt.% nanomaterials. A summary of the density and pH data for the combined solutions is shown in Table 5. 

#### 3.1.5. Core Plugs 

To investigate the effect of rock properties on the recovery process, two outcrop rock types were selected. Berea and Keuper outcrops mimic to a certain extent the potential fields. The main properties are summarized in Table 6. Berea is a yellowish grey sandstone consisting of very homogeneous, well-sorted sand with on average 90% quartz, 4% feldspar, and approximately 6% clays (mainly kaolinite with small amounts of illite and chlorite). Calcite, siderite, and pyrite can be present with quantities <1%. Keuper sandstone is a well-sorted arenite, which main minerals are quartz (97%), clays (~3%), and plagioclase and calcite (both <1%). Accessory minerals are ilmenite, rutile, and goethite. The iron oxides cover the mineral grains and cause the reddish color of the rock. Further information on the outcrops used in this work can be seen in our previous work [10,38].

### 3.2. Experimental Evaluations

All experimental data presented here is reported after at least three separate measurements. This is to ensure repeatability and reproducibility of the observed behavior. Therefore, repeat experiments were performed to ensure that results are reliable. 

#### 3.2.1. Interfacial Tension (IFT) Measurements

IFT measurements were carried out between different EOR fluids and high and low TAN oil in order to investigate the effect of the type of nanomaterial, presence of alkali, and oil composition on interfacial chemistry. IFT was measured using a spinning drop tensiometer (SDT) manufactured by Krüss GmbH (Hamburg, Germany). All measurements were conducted at reservoir temperature (60 °C) using a predefined setting rotational speed of 7000 rpm, which was maintained throughout the measurements. The total observation time was 300 min for each measurement, providing 900 data points with a time interval of 20 s between readings. As reported by Arekhov et al. [38], IFT data is considered as an indexer of the behavior rather than a final value, given that the measurements can be challenging in terms of reproducibility. 

#### 3.2.2. Phase Behavior Tests

The phase behavior was assessed using the procedure described by [9,37]. Graduated glass pipettes with a capacity of 10 mL were filled with the aqueous phase and the oil at a ratio of 1:1. Subsequently, the pipettes were sealed with a CH_4_/O_2_ flame. The pipettes were shaken for 70 h before being placed in the oven at 60 °C. Volumes of each phase (brine, oil, and emulsion) were read for the next 100 days. This time was chosen because it is believed that an equilibrium is reached within this period. Results obtained here are a complement of those presented by Neubauer et al. [9]. We found a good agreement of the phase haviour data and hence a way of validating the results. 

#### 3.2.3. Amott Spontaneous Imbibition

This type of evaluation is commonly used to evaluate the effect of chemical formulations on wettability changes. Once the tested fluid imbibes into an oil saturated core, produced oil is tracked. The experimental setup is simple; the core plugs, which are saturated with oil until irreducible water saturation, are submerged in the displacing fluid. Oil production over time is recorded by measuring the volumes in the graduated pipette. Further information on the procedure can be seen in our previous work [10,38]. Core saturation as well as EOR flooding were performed placing the core in a vertical position. Injecting from bottom to top in order to overcome any possible gravity effects. It should be noted that different combinations/cases were investigated for the spontaneous imbibition tests, in order to capture the various effects/parameters that control this process:

- Brine (Table 2): Softened injection brine (TW) and synthetic formation brine (FW);
- Nanomaterials (Table 4): Type A and type B;
- Oil (Table 3): high total acid number (TAN) and low TAN;
- Rock type (Table 6): Berea and Keuper;
- Alkali: No presence or 3000 ppm Na_2_CO_3_.

### 3.3. Modeling Fundamentals and Approach 

#### 3.3.1. Capillary Diffusion Coefficient 

The amount of mixing in a miscible displacement process is often assessed by the variable molecular diffusion coefficient [51]. The capillary imbibition can be approached as diffusion mechanism after neglecting the convection and gravity effects [47].

(1) $$\Phi \frac{\delta S_{w}}{\delta t}=-\nabla \left[k\frac{k_{W}k_{o}}{k_{t}}\nabla P_{c}\left(S_{w}\right)\right]\mathrm{with}\;:k_{o}=\frac{k_{ro}}{\mu_{o}},\;\mathrm{and}k_{w}=\frac{k_{rw}}{\mu_{w}}\;k_{t}=k_{o}+k_{w}$$

where k is the single-phase permeability, *S* is the saturation, *µ* is the viscosity, and *o* and *w* stand for the oil and phase, respectively.

After introducing the imbibition capillary pressure *P_c_*(*S_w_*), Equation (1) can be expressed by the means of the capillary diffusion coefficient (*D_c_*) as follows:

(2) $$\frac{\delta S_{w}}{\delta t}=D_{c}\Delta \left(S_{w}\right)$$

In a cylindrical geometry, the oil saturation can be normalized as follows:

(3) $$S_{o}^{*}=\frac{S_{o}-S_{of}}{S_{oi}-S_{of}}$$

*So∗* can be also expressed as a product of two solutions: *C_ps_* for a plane sheet and *C_cyl_* for a cylinder as follows:

S_o_^∗^ = C_ps_ C_cyl_ (4)

Including diffusion proposed by Crank [52].

(5) $$\begin{array}{c} C_{ps}=\sum_{n=1}^{\infty }\frac{8}{{\left(2n+1\right)}^{2}}exp\left(-D_{c}{\left(2n+1\right)}^{2}\pi^{2}\frac{t}{4l^{2}}\right)\\ C_{cyl}=\sum_{n=1}^{\infty }\frac{4}{r^{2}q_{n}{}^{2}}exp\left(-D_{c}q_{n}{}^{2}t\right) \end{array}$$

where, *l* is the length of the plug, *r* the radius, and *q_n_* are the positive roots of the equation: *J_o_*(*rq_n_*) = 0, *J*_0_ is the zero order of the first type Bessel’s function. By fitting the normalized oil saturation values obtained into Equation (4), a constant *D_c_* can be estimated using a *Non-Linear Least-Squares* fitting algorithm.

#### 3.3.2. Inverse Bond Number

IFT reduction in EOR operations impacts the capillary trapping number. However, in a mere spontaneous imbibition, the flow is governed by capillary pressure devoid of viscous pressure gradient. In this case, gravity forces can play an important role. One example is naturally fractured reservoirs, where the matrix height and the vertical connectivity are the key answer to the question of which recovery mechanism will be dominant [53]. The relationship between gravity and capillary force is best described by inverse Bond number *N_B_*^−1^. The inverse Bond number represents the ratio of capillary to gravity forces and can be calculated by Equation (6).

(6) $$N_{B}^{-1}=C\frac{\sigma \frac{\bar{\varphi }}{k}}{\Delta \rho \delta H}$$

where *C* = 0.4 for a capillary tube model, *H* is the core height [meter], *σ* is the interfacial tension [mN m^−1^], *φ* is the porosity [%], *k* is the absolute permeability [mD], and *∆ρ* is the density difference between the phases [kg/m^3^]. 

The impact of IFT reduction in EOR applications is commonly addressed by analyzing the capillary number which relates to the ratio of viscous to capillary forces. However, during the spontaneous imbibition process, the gravity forces play an important role alongside the capillary forces. The gravity forces are mainly governed by the density contrast and the height of the matrix block. The density contrast between phases triggers gravity drainage, which can be translated into a lower contribution by capillary forces (N_B_^−1^ is low). This ratio can be affected by changing matrix height or reducing the IFT.

The dynamics of spontaneous imbibition can be described by imbibition rate and ultimate recovery, which are influenced by changes in the inverse bond number [54,55]. An increasing inverse Bond number will lead to higher ultimate recovery due to weaker capillary forces. However, since the spontaneous imbibition is a capillary driven process, imbibition rate will be lower [54]. This was later attributed to the fact that low IFT increases the imbibition rates at late time or to wettability alteration process [55]. Imbibition rate is only affected in ultra-low IFT systems. 

When IFT ranges between high and intermediate values, the imbibition rate is not influenced by IFT [53]. When IFT is reduced enough to enable emulsification, the solubilization factor can be used to correlate the recovery using SI and the emulsion formed (Chen et al. [56]). The solubilization factor SF can be computed by incorporating the oil solubilization ratio *SP*_o_ and the water solubilization ratio *SP_w_* into the Equation (7) (Sheng [37]).

(7) $$S_{F}=\sqrt{SP_{w}^{2}+}SP_{o}^{2}=\sqrt{{\frac{V_{w}}{M_{s}}}^{2}}+{\frac{V_{o}}{M_{s}}}^{2}$$

where *SP_W_* and *SP_O_* are water and oil solubilization ratios, mL g^−1^; *V_W_* and *V_O_* are the volume of water and oil in the emulsion, mL; and *M_S_* is the weight of the surfactant agents, g. Since the inverse Bond number as expressed in Equation (6) does not take the wettability factor into account, other expressions of the inverse Bond number were developed for instance by Babadagli [55], to include the wettability-related parameter *f* (θ) as shown in Equation (8).

(8) $$N_{B}^{-1}=C\frac{f\left(\theta \right)\sigma \sqrt{\frac{\bar{\varphi }}{k}}}{\Delta \rho \delta H}$$

## 4. Results and Discussion

### 4.1. Interfacial Tension (IFT) 

#### 4.1.1. High TAN Oil

**Figure 3:**
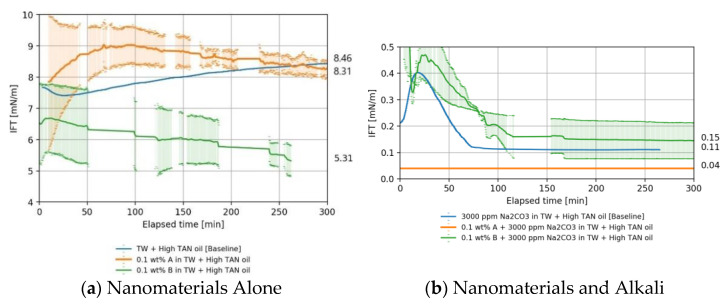
Interfacial tension (IFT) versus time measured between high TAN oil and brine, nanomaterials, and alkali (60 °C, 7000 rpm). The IFT between brine (TW) with and without addition of 0.1 wt.% nanomaterials (type A and B) is shown in (**a**). Nanomaterials in a solution with alkali are shown in (**b**).

Figure 3 presents the IFT behavior obtained for solutions with and without alkali when in contact with high TAN oil. The IFT between oil and brine was measured at around 8.46 mN/m. The reported value differs slightly from the value published by Arekhov et al. [38] for crude oil from the same reservoir. Note that the authors also reported high deviations in the IFT data. The findings for each case are discussed below in more detail.

##### Nanomaterials as a Standalone EOR Agent in High TAN Oil

As shown in Figure 3a, nanomaterial type B could lower the IFT compared to the base case (softened injection brine, TW) by 1.5 times. The IFT reduction is attributed to adsorption of particles onto the oil–water surface. IFT behavior depicted a dependency on surface modification. The diol-modified nanomaterial type B could lower the IFT by a factor of 1.5 to 5.3 mN/m (Figure 3a), while the impact of the PEG-coated nanomaterial type A on IFT was negligible (8.3 mN/m). In general, IFT reduction by nanomaterials is attributed to adsorption of particles to the oil–water interface. According to Daghlian et al. [27] the adsorption is limited by the hydrophobicity of the particle surface. Hydrophilic particles tend to remain suspended within the aqueous phase instead of traveling towards the interface. Nanomaterial B is more negatively charged; thus, the electrostatic repulsion becomes more effectual as reported by Vatanparast et al. [57]. With nanomaterial B, the surface was modified to increase its hydrophobicity. 

##### Nanomaterials and Alkali in High TAN Oil

The utilization of alkali leads to a drastic reduction of IFT as shown in Figure 3b. As reported by Schumi et al. [39] for the same oil, alkali reacts with the acidic components of the oil, producing in situ surfactants (soaps) which contribute mainly to the IFT reduction. An interesting behavior was a slight increase in the IFT at the beginning of the measurement, followed by a steep drop and stabilization—refer to Figure 3b between the elapsed time of 0 and 80 min. Neubauer et al. [9] observed a similar behavior for the tested oil, and such behavior was also explained by Sharma et al. [58]. The latter refer to that as the shielding of the newly formed surfactants at the interface, leading to a deceleration of the alkali consumption. Due to convective diffusion at the interface, surfactant molecules migrate towards the aqueous phase, allowing the reaction to occur until an equilibrium has been reached. 

In comparison with the base case, TWe with 3000 ppm of Na_2_CO_3_ shown in Figure 3b blue, nanomaterial B had no substantial effect on the interfacial tension (0.15 vs. 0.11 mN/m). This can be explained by the very prominent electrostatic repulsion between the anionic surfactant molecules and silica particles [57]. However, surfactant molecules are the main player in IFT reduction. As reported by Huh et al. [28], when nanomaterials replace the surfactant molecules at the interface, the IFT is less reduced. However, a synergistic effect between nanomaterial type A and the alkali was observed (Figure 3b, orange line), resulting in IFT reduction of 2.7 times compared to alkali alone (blue line). 

#### 4.1.2. Low TAN Oil

**Figure 4:**
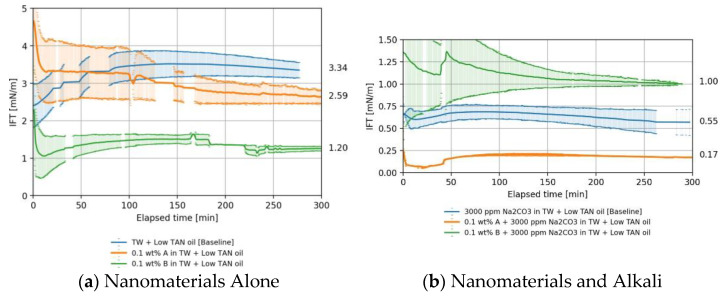
Interfacial tension (IFT) versus time measured between low TAN oil and brine, nanomaterials, and alkali (60 °C, 7000 rpm). IFT between brine (TW) with and without addition of 0.1 wt.% nanomaterials (type A and B) is shown in (**a**). Nanomaterials in a solution with alkali is shown in (**b**).

Figure 4 presents the IFT behavior obtained for solutions with and without alkali when contacting the low TAN oil. The IFT for the oil/brine system was around 3.0 mN/m (Figure 4a, blue line). This value is within the range of 2 to 4.5 mN/m reported by Arekhov et al. [38].

##### Nanomaterials as a Standalone EOR Agent in Low TAN Oil

The addition of nanomaterial type A resulted in a slight IFT reduction of 1.26 times compared to the base case from 3.3 to 2.60 mN/m (refer to Figure 4a comparing blue and orange lines). Nanomaterial B performed somewhat better and reduced the IFT by a factor of 2.75, from 3.3 to 1.20 mN/m, supposedly due to the more negatively charged surface as previously explained. IFT reduction is attributed here to the accumulation of particles at the fluid/fluid interface.

##### Nanomaterials and Alkali in Low TAN oil

The addition of alkali to TW brine (18.96 g/L NaCl and 1.96 g/L NaHCO_3_) had a significant impact on the IFT despite the low TAN number of the oil (IFT reduction from 3.34 to 0.55 mN/m, blue line in Figure 4b). As in the high TAN case, nanomaterial type A outperformed type B when combined with alkali, see Figure 3 for additional comparison.

The addition of nanomaterial B did not result in further IFT reduction (see Figure 4b, green line). The IFT is mainly driven by the reaction between alkali and acids in the crude oil. Nanomaterial A exhibited a strong synergy with alkali, and IFT was lowered to 0.17 mN/m as shown in Figure 4b, orange line. This is a factor of 3.2 compared to the baseline (blue). This can be explained by the more neutral surface charge of the PEG coating as discussed in chapter 4. The reaction between alkali and acidic components of the oil takes place, but the oil does not contain enough acids to sustain the reaction and lower the IFT further. Therefore, the IFT was reduced to lower values for high TAN oil than for low TAN oil.

#### 4.1.3. Overall IFT Findings

Nanomaterials were effective in terms of IFT reduction when combined with alkali as observed in Figure 3 and Figure 4. IFT values as low as 0.04 mN/m can be achieved, which can be deemed a very stable equilibrium IFT. Table 7 provides a summary of the average values obtained for various measurements. IFT values need to be referenced to baseline values in order to evaluate the effects of chemicals on IFT. For oil–brine systems with high IFT, the measurements have large standard deviations. Thus, the measurements of high IFT cases cannot offer a conclusive quantification but rather a qualitative comparison of the IFT trends.

The modest IFT reduction with the PEG-coated nanomaterial type A suggests that the nanomaterials were unable to significantly enhance the interaction between the immiscible oil and water phase. The observation agrees with evaluations presented by Sharma et al. [16], Ahmed et al. [17], and Bila et al. [31], who only observed slight changes of the IFT between oil and brine when nanomaterials were added. Moreover, despite the fact that both nanomaterials have the same base composition (SiO_2_) and approximately the same dimensions, the diol-coated nanomaterial B performed better than type A in terms of IFT reduction. Note that the case of study is the proposal of an effective EOR formulation with the synergistic effects of nanomaterials and alkali. We found here that, under the testing conditions, nanomaterials in combination with alkali (Na_2_CO_3_) substantially reduced the IFT for high TAN oils. 

### 4.2. Phase Behavior Observations

In order to evaluate the effects of divalent cations on the EOR process, synthetic formation brine (FW, composition shown in Table 2) was used in addition to synthetic softened brine (TW). During fluid preparation, white sediment deposited in the solutions that contained nanomaterials and alkali, which were prepared using synthetic formation brine rich in divalent cations (TW). Nevertheless, this precipitation did not occur in the nanomaterial alone diluted with formation brine (FW). A solution of 3000 ppm Na_2_CO_3_ was prepared using formation brine and monitored for a few hours. We believe that as reported by Spanos and Koutsoukos [59], the precipitation is triggered by the reaction of dissolved calcium with alkali due to supersaturation of calcium carbonate at a specific pH range. In the presence of nanomaterials, hetero aggregation of calcium carbonate flocs and the nanomaterials (Liesegang et al. [60]) or incorporation of nanoparticles into calcium carbonate (Magnabosco et al. [61]) is believed to occur. Hence, we did not further test FW in the presence of the nanomaterials and alkali since it is not recommended to use alkali when the formation brine is rich in divalent cations.

**Figure 5:**
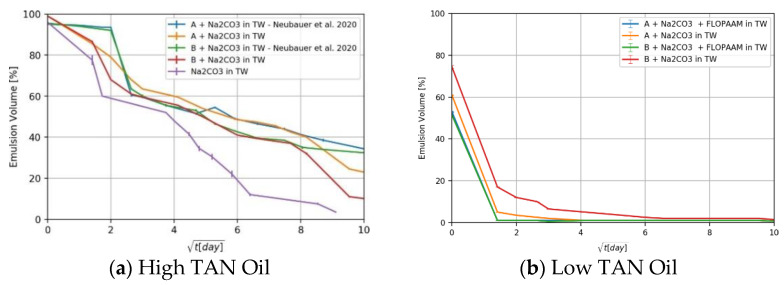
Phase behavior results represented as emulsion volume versus square root of time for the nanomaterials dissolved in TW. The case of high TAN oil is shown in (**a**), and the case of low TAN oil is shown in (**b**). Note that reported data is averaged from triplicate samples.

For the high TAN oil it was observed that the emulsification process was mainly driven by the alkali acid reaction (Figure 5a). Nanomaterials alone were not able to emulsify a significant amount of oil. However, a thin emulsion layer was observed at the oil/brine interface, and it was stable throughout the measurement period. Figure 5a shows that the emulsions formed under the presence of nanomaterials and alkali were more stable compared to alkali alone (purple line). For the low TAN oil Figure 5b, high emulsion volumes were observed directly after mixing but failed to remain over time. Although some effects were observed by the use of nanomaterial B, Figure 5b red line, the changes do not lead to proper conclusions. 

In previous work for the same oil from Neubauer et al. [9], nanomaterial B produced more stable emulsions, which had also better rheological properties after 100 days. We attribute the differences to: (1) the initial energy input to the experiment, which was also reported by Arab et al. [19] and (2) possible error on the visualization/readings. 

**Figure 6:**
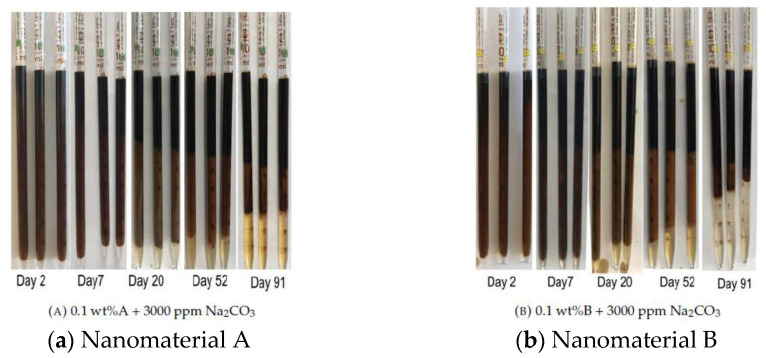
Images of phase behavior results represented as visualization of formed emulsions over time for the nanomaterials dissolved in (18.96 g/L NaCl and 1.96 g/L NaHCO_3_) and alkali. The case of nanomaterial A is shown in (**a**), whereas the case of nanomaterial B is shown in (**b**). Note that reported data is presented from triplicates.

To further elaborate on the observations for high TAN oil, Figure 6 depicts an example of some of the pipettes evaluated. For instance, Figure 6b at day 52 shows the middle phase emulsion. It should be noted that the emulsions obtained with nanomaterial B have a slightly darker color, which indicates higher oil content. To explain that, two questions have to be answered. First, does phase separation occur? And second, how are emulsions stabilized? The main mechanism of phase separation is believed to be associated with droplets junction. Emulsions are created when oil droplets are coated with a hydrophilic layer and dispersed within the aqueous phase. Binks and Rodrigues [21] suggested that emulsions are stabilized by nanomaterials when the oppositely charged surfactant molecules are attached to nanoparticles surface changing their wettability. In our case, the oil droplets, which are covered by anionic surfactants (soaps), are sterically hindered from coagulation due to the electrostatic repulsion. We believe that the dispersion of negatively charged particles in the medium enhances this repulsive interaction.

In addition, as further input for the modelling the Solubilization Factor (SF) was computed (using Equation (7)) for the solutions containing nanoparticles only, and they were treated as surfactant molecules. This was not applicable to alkaline solutions since the mass of generated soap is unknown. After 100 days SF using nanomaterial A was 13.3, while for B it was 35.2.

### 4.3. Amott Spontaneous Imbibition Evaluations

#### 4.3.1. Baseline Experiments—Softened Brine

**Figure 7:**
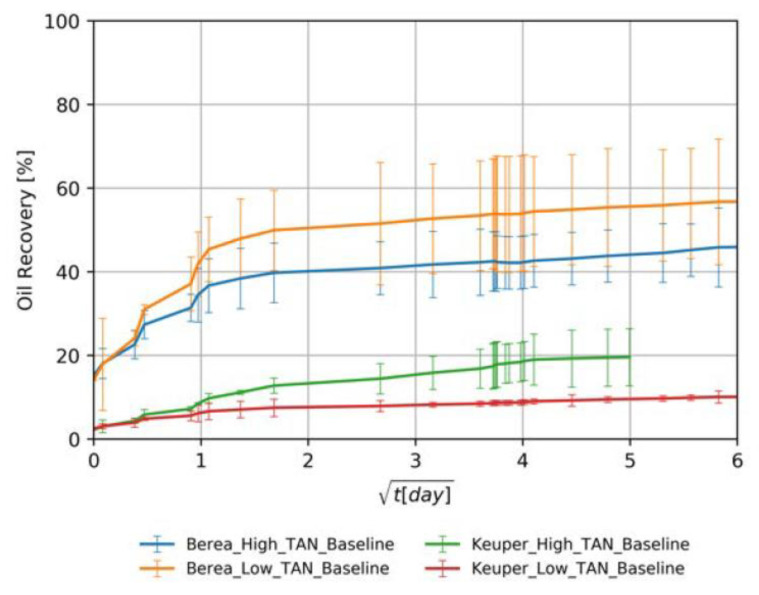
Oil recovered by the imbibition of TW (18.96 g/L NaCl and 1.96 g/L NaHCO_3_) in cores saturated with high or low TAN. The data is shown over the square root of time to define the maximum achieved oil recovered.

The baseline experiments consisted of four different oil/rock combinations soaked in softened injection brine. These tests are crucial to assess the additional recovery by utilizing EOR fluids. Figure 7 shows the oil recovery versus square root of time. The data/observations serve as guidance to interpret subsequent results presented in the section:

- Initial wettability state: The high recoveries from Berea samples for both oil types (orange and blue lines in Figure 7) can be attributed to a mixed-wet state. The recovered percentages for Berea and Keuper are consistent with the values reported by Neubauer et al. [10]. However, values obtained from the Keuper samples differ from Arekhov et al. [38]; we attribute it to a possible lack of consistency on core the saturation process.
- Core plugs permeability: Since the spontaneous imbibition is a capillary-driven mechanism, the recovery was higher/faster in cores with lower permeability (Berea, orange and blue lines in Figure 7) where the capillary forces tend to be more significant. This is in line with the behavior proposed by Morrow et al. [62].
- Visual observation of oil droplets: We observed that oil droplets were relatively large in volume (big oil droplets), which indicates a high IFT regime between the oil and brine. This observation was in accordance with the IFT data presented in Section 4.1.
- Effects of oil viscosity: There was a clear contrast of oil viscosities, with low TAN oil having significantly lower viscosity than high TAN oil (6 mPa∙s and 11.90 mPa∙s, respectively, refer to Table 3). In core plugs with low permeability, recovery values were higher for the low TAN oil (Berea, orange, and blue lines in Figure 7). Similar observations were reported by Schechter et al. [54], Zhou et al. [63] and Meng et al. [64], where high recovered oil was observed in low-permeability dolomite. The authors attributed that to the proportionality between ultimate oil recovery to the square root of dimensionless time, where wettability is highly dependent on IFT and the mobility ratio.
- Clay content in Keuper samples: Core plugs with large mineralogical heterogeneity like the Keuper cores containing goethite are believed to be more oil wet [65,66]. Sayyouh et al. [65] suggested that under low to moderate salinity the tendency towards oil-wet increases with increasing clay content. More oil wetness makes the water less favorable to imbibe the core, therefore recovery under spontaneous imbibition would be lower.

#### 4.3.2. Influence of the Nanomaterials as a Standalone EOR Agent

##### Berea Core Plugs

**Figure 8:**
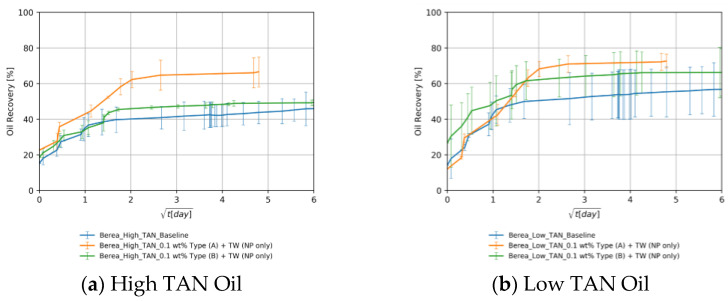
Oil recovered in Berea core plugs by the imbibition of nanomaterials alone dissolved in TW (18.96 g/L NaCl and 1.96 g/L NaHCO_3_) in cores saturated with high TAN oil (**a**) and low TAN oil (**b**). The data is shown over the square root of time to define the maximum achieved oil recovered.

The utilization of nanomaterials resulted in additional recoveries of up to 20% for both oil types, compared to the baselines (blue in case Figure 8a,b). As shown in Figure 8a orange line, nanomaterial A could achieve a higher recovery than B. This can be explained by the more neutrally charged surface of the PEG-coated nanomaterial A, discussed in the previous sections. In addition, the solubilization factor SF is lower for nanomaterial A than for B (13.3 vs. 35.2). This agrees with Chen et al. [56], who suggested that a lower solubilization factor would lead to higher recovery under spontaneous imbibition. Furthermore, recoveries were higher for low TAN oil than with high TAN oil (Figure 8b vs. Figure 8a). This can be explained by the lower viscosity of the low TAN oil. The lower IFT, observed for low TAN oil using nanomaterial A, could also contribute to the higher/faster oil recovery for nanomaterial A as compared to the one using B.

##### Keuper Core Plugs

**Figure 9:**
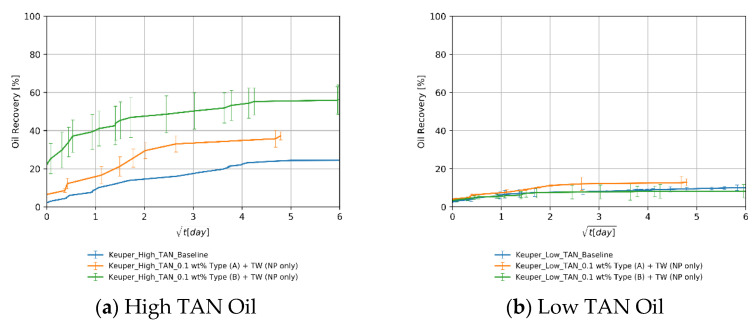
Oil recovered in Keuper core plugs by the imbibition of nanomaterials alone dissolved in TW (18.96 g/L NaCl and 1.96 g/L NaHCO_3_) in cores saturated with high TAN oil (**a**) and low TAN oil (**b**). The data is shown over the square root of time to define the maximum achieved oil recovered.

Figure 9 shows that the contribution of nanomaterials to additional recovery varied depending on the nanomaterial type and oil used. One can observe, for instance, from Figure 9a that nanomaterial B (green line) was more effective with high TAN oil in terms of recovery in contrary to what was observed for Berea core plugs. This confirms the premise proposed by Omurlu et al. [67] that the more negatively charged, diol-modified nanomaterials tend to adhere better to the pore walls that are coated by ferric oxide, which promotes more positive surface charge. The observation is also in agreement with Alvarez-Berrios et al. [30], who proposed that due to this adhesion (NPs adhering to the rock surface), the mineral surface will be tuned towards more favorable wettability state. One reason to explain this effect is the exchange of the nanofluid forms a wedge-shaped thin film due to the existence of disjoining pressure. Figure 9b indicates that the recoveries with low TAN oil were lower than the ones obtained with high TAN oil. Nanomaterial A could achieve slightly higher recovery than B, which did not show any improvement compared to the baseline. This was previously explained and attributed to the more neutral surface charge of nanomaterial A. The recovery mechanisms in Keuper are more complicated due to the presence of clay minerals and iron hydroxides, discussed in detail in Section 4.3.1.

#### 4.3.3. Influence of the Oil—TAN

**Figure 10:**
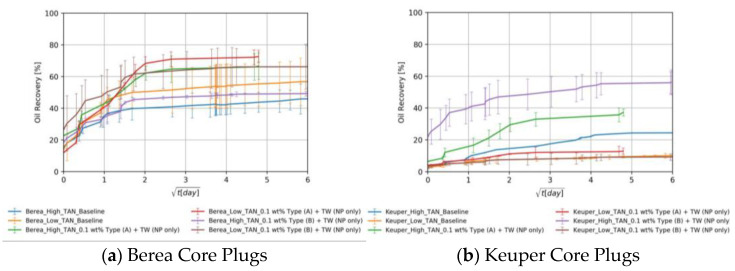
Oil recovered in Berea (**a**) and Keuper (**b**) core plugs by the imbibition of nanomaterials alone dissolved in TW (18.96 g/L NaCl and 1.96 g/L NaHCO_3_) in cores saturated with the high or low TAN oil. The data is shown over the square root of time to define the maximum achieved oil recovered.

Figure 10 depicts an overall comparison of the behavior obtained for the different oils in Berea and Keuper plugs. On the one hand, Figure 10a demonstrates that for all aqueous solutions that do not contain alkali, the recoveries with low TAN oil were higher than the ones with high TAN oil. This may be due to two reasons: (1) the lower viscosity of low TAN oil, discussed in detail in Section 4.3.1 and (2) in agreement with Buckley [68], the higher asphaltene and polar components content in high TAN oil (2% vs. 1%) promotes more oil wetness. 

On the other hand, Figure 10b shows the opposite behavior observed for Keuper cores, i.e., higher recoveries for high TAN oil than for low TAN oil. Similar observations were made by Meng et al. [64] for high permeable cores. Due to the high permeability of Keuper core plugs, oil viscosity does not play a major role in the spontaneous imbibition process; subsequently, the capillary forces are also weaker.

#### 4.3.4. The Effect of Divalent Cations

**Figure 11:**
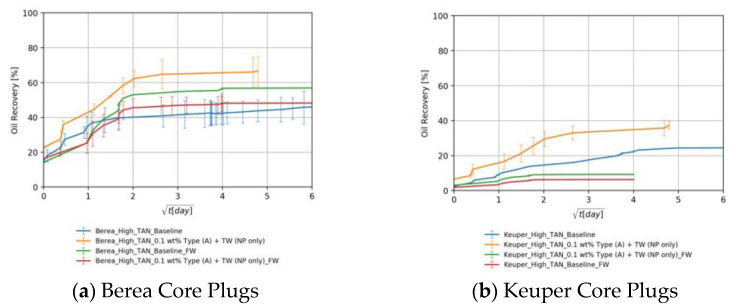
Oil recovered in Berea (**a**) and Keuper (**b**) core plugs by the imbibition of nanomaterials alone dissolved in TW in cores saturated with high TAN oil and initial water saturation using FW. Data is shown over the square root of time to define the maximum achieved oil recovered.

Cores of both rock types were pre-saturated with synthetic formation brine (FW), which is rich in divalent cations. However, EOR fluids were prepared using synthetic injection brine (TW). Figure 11a shows that, in the baseline for Berea, recoveries were slightly higher in the core plugs saturated with formation brine (58.3% of initial oil saturation). Nevertheless, nanomaterials had a negative impact on the recovery in the presence of divalent cations (49±3% of % of initial oil saturation). The results are in alignment with Metin et al. [69], who investigated the effect of divalent cations on colloidal stability. Divalent cations like calcium or magnesium may trigger the aggregation of silica nanomaterials. This could result in pore throat plugging and formation damage. Figure 11b shows that Keuper core plugs saturated with formation brine achieved lower recovery values than when they were pre-saturated using TW (6.3% vs. 9.2% of the percentage of initial oil saturation). This could be explained by the fact that the presence of divalent cations promotes more oil wetness [70]. In Keuper cores, the use of nanomaterials in EOR fluids succeeded in slightly enhancing the recovery. The larger pore throats in the Keuper cores and the higher clay content and mineralogical heterogeneity that involves mechanisms that are more complex can also explain these observations.

#### 4.3.5. The Effect: Nanomaterials and Alkali 

##### Berea Core Plugs

**Figure 12:**
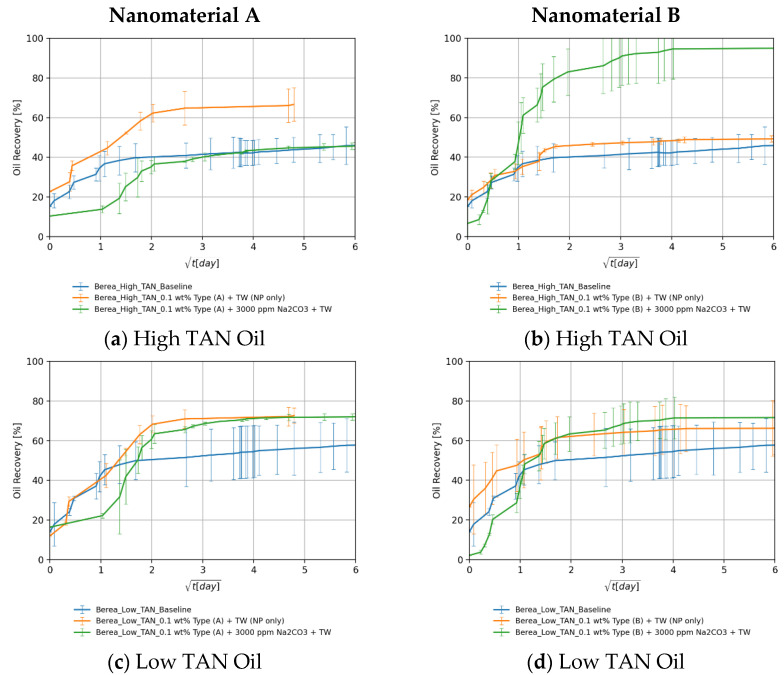
Oil recovered in Berea core plugs by the imbibition of nanomaterials in synergy with alkali dissolved in TW (18.96 g/L NaCl and 1.96 g/L NaHCO_3_) in cores saturated with high TAN oil (**a**,**b**) and low TAN oil (**c**,**d**). Data for nanomaterial A is shown in (**a**,**c**), whereas for nanomaterial B it is shown in (**b**,**d**). Data is shown over the square root of time to define the maximum achieved oil recovered.

Figure 12a,b show that alkali per se boosted the recovery by 23%. This can be explained by the acid–alkali reaction and the generation of in-situ surfactant. The combination of alkali and nanomaterial B resulted in a significant improvement in recovery. The high imbibition rate with high ultimate recovery suggests a strong rock fluid interaction. Dean–Stark extraction (refer to Appendix A for specific details) revealed that the imbibed water volume is lower than monitored during the Amott test, which supports the assumption that the volume produced is a macroemulsion. This can be linked to the low IFT, which facilitates spontaneous emulsification. It also explains some abnormally high recoveries (>100% of initial oil saturation) in some core plugs. The capillary diffusion coefficient, explained later on, was reduced in this case by a factor of 5% (9.38 × 10^−9^ vs. 9.88 × 10^−9^ for the baseline). However, the IFT was 19-times lower (0.15 vs. 8.40 mN/m). This suggests that a major wettability alteration has taken place as also reported by Chevalier et al. [47]. PEG-coated nanomaterial type A combined with alkali did not improve the ultimate recovery despite the significant IFT reduction. The imbibition rate was low in this case due to the substantially low IFT. Nevertheless, the ultimate recovery falls close to the baseline.

For the low TAN oil shown in Figure 12c,d, it can be seen that despite the positive impact of alkali and nanomaterials on IFT as well as on emulsion stabilization, their use did not result in a recovery enhancement. The highest ultimate recovery was achieved using alkali alone (86.8% of initial oil saturation). Since the enhancements observed during fluid–fluid interactions did not result in additional recovery, we can attribute that to a major rock–fluid interaction. The synergy between alkali and nanomaterials that was observed in in the experiments using high TAN oil and Berea cores was imperceptible. Overall, Table 8 summarizes all ultimate recoveries (as a percent of initial oil saturation) from Berea core plugs using the different EOR formulations.

##### Keuper Core Plugs

**Figure 13:**
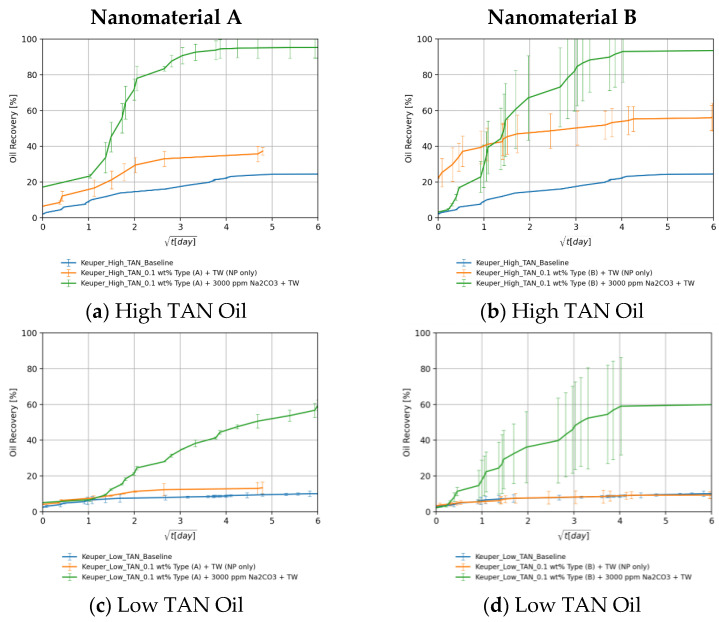
Oil recovered in Keuper core plugs by the imbibition of nanomaterials in synergy with alkali dissolved in TW (18.96 g/L NaCl and 1.96 g/L NaHCO_3_) in cores saturated with high TAN oil (**a**,**b**) and low TAN oil (**c**,**d**). Data for nanomaterial A is shown in (**a**,**c**), whereas for nanomaterial B it is shown in (**b**,**d**). Data is shown over the square root of time to define the maximum achieved oil recovered.

For the high TAN oil shown in Figure 13a,b, there was a significant increase in ultimate recovery after introducing alkali to the EOR fluid (green lines). Alkali reaction with acidic components and the generated soap is responsible for the IFT reduction seen in Figure 3b and, consequently, for the high ultimate recovery. In some cases, the ultimate recovery exceeded 100% of initial oil saturation, which is attributed to the spontaneous emulsification of the oil. This was confirmed by Dean–Stark extraction (refer to Appendix A for specific details) since the extracted water volume was less than the recorded imbibed volume added to the initial water in place. It became difficult to evaluate the role of nanomaterials because the recoveries with alkali fall close to each other. For this purpose, further investigations should be carried out (contact angle measurements, core floods, etc.).

For the low TAN oil shown in Figure 13c,d, the utilization of alkali enhanced the recovery as seen in the green lines. After the nanomaterials were introduced into the alkaline EOR fluid, the recovery was significantly boosted. This is a very good indicator of the synergy between alkali and nanomaterials. Some of these synergistic effects were observed during fluid–fluid interactions (Section 4.1). PEG-coated nanomaterial type A performed better due to the neutral surface charge and the affinity of clay to adsorb SiNP coated with more neutral polymers, as also demonstrated by Omurlu et al. [67]. Overall, Table 8 summarized all ultimate recoveries (as a percent of initial oil saturation) from Keuper core plugs using the different EOR formulations.

### 4.4. Modeling Results 

#### 4.4.1. Shape of the Recovery Curve

**Figure 14:**
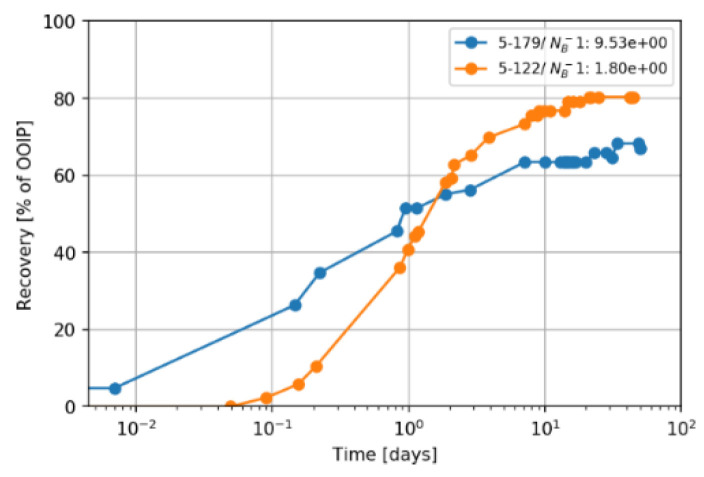
A comparison of the recovery curve for different values of the inverse Bond number, *N_B_*^−1^. The example shows two Berea core plugs (5-179 and 5-122) saturated with low TAN oil recovered by the imbibition of TW (18.96 g/L NaCl and 1.96 g/L NaHCO_3_) in core 5-179 and the imbibition of a mixture (0.1 wt.% Type B + 3000 ppm Na_2_CO_3_ + TW) in core 5-122.

Figure 14 shows the ultimate recovery curve of two Berea cores saturated with low TAN oil. Core 5-179 submerged in softened injection brine (TW), while core 5-122 submerged in alkali and nanomaterials (0.1 wt.% Type B + 3000 ppm Na_2_CO_3_ + TW). Since the IFT was reduced, the recovery was slower (lower imbibition rate), whereas a rapid imbibition can be observed in the case of high IFT (5-179). Lowering IFT weakens the capillary forces that are significant for counter-current imbibition, observed during baseline experiments. In agreement with the observations by Al-Quraishi et al. [53], gravitational forces are more effective in the case of a low inverse Bond number, which results in sufficient hydrostatic pressure behind oil droplets to detach them before snap-off takes place. This in turn makes gravitational forces significant for concurrent spontaneous imbibition.

#### 4.4.2. Recovery vs. Inverse Bond Number (N_B_^−1^)

**Figure 15:**
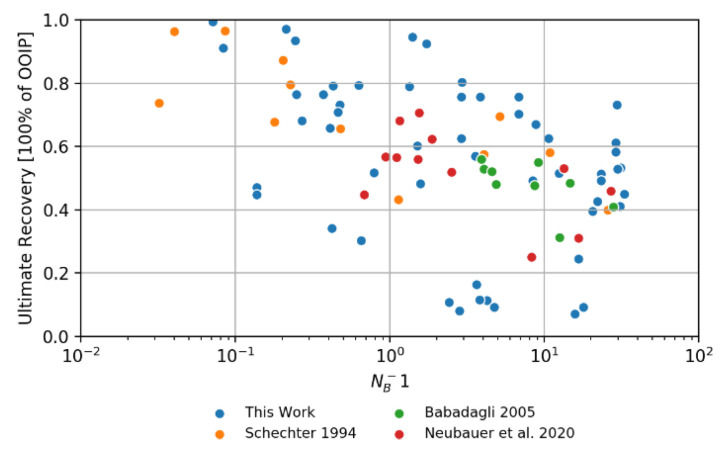
Comparison of oil recovered versus inverse Bond number, *N_B_*^−1^ (using Equation (6)). Data is shown for the findings of this work and selected literature data.

In Figure 15 we show an interesting comparison using the data obtained in this work for Berea core plugs (permeability around 400 mD) with the work reported by Neubauer et al. [10] (same rock type and permeability), Schechter et al. [54] (permeability around 700 mD), and Babadagli [55] (SS, permeability around 400 mD). These authors also investigated low IFT imbibition. There is a general trend showing that the ultimate recovery increases when the inverse Bond number *N_B_*^−1^ decreases. All plotted data seems to be in keeping with our findings. However, no fully systematic trend can be recognized since the data is obtained from different oil systems. 

**Figure 16:**
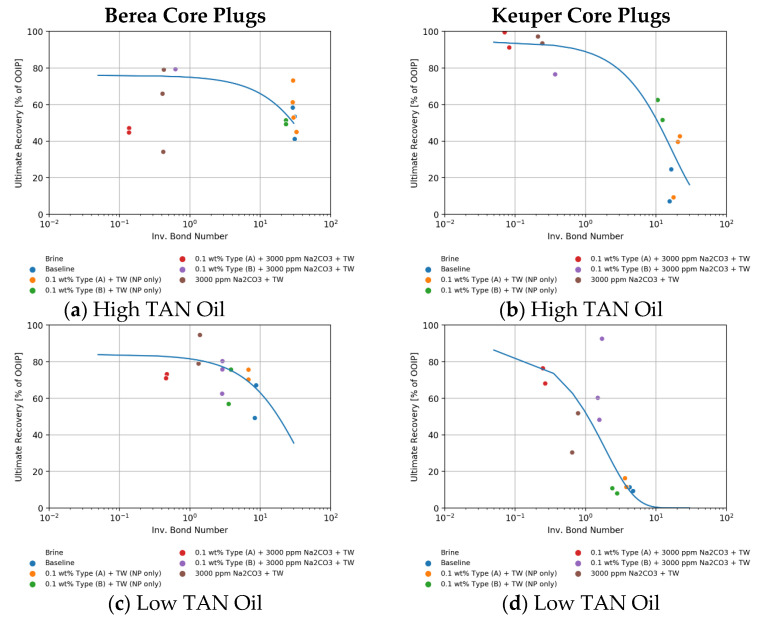
Ultimate recovery in % versus inverse Bond number, *N_B_*^−1^ (using Equation (6)) for the different cores (Berea and Keuper). Berea core plugs data is shown in (**a**,**c**) for high and low TAN, respectively. Keuper core plugs data is shown in (**b**,**d**) for high and low TAN, respectively.

Furthermore, in Figure 16, we show a detailed view of the data obtained for each case (rock and oil type). With the exception of the Berea high TAN oil case (Figure 16a), an exponential relationship delivered the best fit. The defined fit agrees with the ones reported by Neubauer et al. [10] and Babadagli [55]. It is worth noting that this analysis does not take the wettability effect into account. Hence, as pointed out by Babadagli [55], in cases where IFT was lowered and the imbibition rate increased, a wettability alteration process must have taken place. A modified inverse Bond number, which includes wettability by using contact angles, was calculated for some select cases in which contact angles were available (Appendix C). For Keuper plugs which are oil wet and wettability alteration therefore plays an important role, fits for ultimate recovery versus inverse Bond number were greatly enhanced. 

#### 4.4.3. Capillary Diffusion Coefficient—An Example

**Figure 17:**
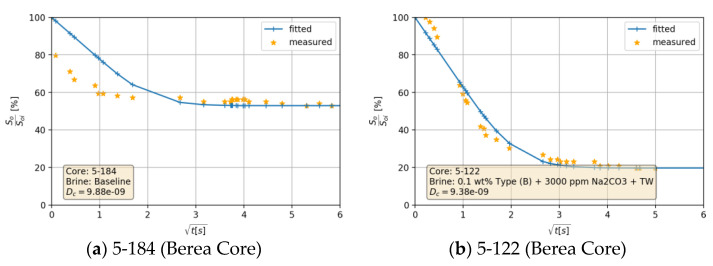
Relative oil saturation vs. square root of time for selected Berea core plugs. Imbibition process taken place is shown in (**a**) for the baseline (TW, 18.96 g/L NaCl and 1.96 g/L NaHCO_3_), whereas the imbibition taking place for the mixture (0.1 wt.% Nano B + 3000 ppm Na_2_CO_3_ + TW) is shown in (**b**).

By fitting the obtained normalized oil saturation values into Equation (4), a constant diffusion coefficient *D_c_* can be estimated using a Non-Linear Least-Squares fitting algorithm. Figure 17 shows that with a constant capillary diffusion coefficient a reasonable fit has been achieved. Mismatches occurred mainly due to the shaking process and spikes in read-off values. For this example, the core 5-184 was submerged in softened injection brine (baseline, Figure 17a), while the core 5-122 was submerged in brine containing 0.1 wt.% nanomaterial type B and 3000 ppm of Na_2_CO_3_ (Figure 17b). Looking back at Table 7, one can observe that IFT was reduced by 98%. Under a fixed wettability state, imbibition kinetics should be lowered by the same magnitude. However, the capillary diffusion coefficient in 5-122 is slightly lower than in 5-184. IFT reduction suggests a weaker capillarity and thus slower imbibition.

Furthermore, analyses of the wettability and diffusion multiple calculations were performed for the inverse Bond number (see Appendix B for details). As mentioned earlier, *N_B_*^−1^ is insensitive to wettability changes. In the case of a stable wettability state, a reduction in IFT should result in slower imbibition and thus a reduction in *D_c_* of the same magnitude. The reduction in *D_c_* can be seen only in the cores submerged in 0.1 wt.% Type B + 3000 ppm Na_2_CO_3_ + TW. However, for the other EOR fluids, imbibition kinetics were accelerated despite the IFT reduction. This can be attributed to wettability alteration towards water wet, which results in rapid imbibition of the aqueous phase into the core. 

Other work that investigated spontaneous imbibition using nanomaterials, for instance Wang et al. [34], also reported an increase in the imbibition rate. Based on data, we consider that nanomaterial A induced an acceleration of the imbibition (in the case of Berea with high TAN oil, *D_c_* was increased by 62% using nanomaterial A compared to 57% with nanomaterial B). By adding 3000 ppm of Na_2_CO_3_, IFT was lowered. Consequently, imbibition kinetics were decelerated compared to the case of nanomaterials only. However, *D_c_* is still higher than the base case except the combinations with nanomaterial B.

## 5. Conclusions

We observed a positive impact of nanomaterials on many EOR-related parameters. Once combined with alkali, this impact was more significant and cannot be solely attributed to one effect but rather a firm synergy. Therefore, based on the conditions evaluated here, we demonstrated that the formulation nanomaterial alkali could bring benefits. 

We observed that surface modification of the particles is a determinant factor in the recovery process. A PEG-coated, near neutrally charged surface was more effective in IFT reduction once combined with alkali, whereas a more negatively charged surface performed better as a standalone. During spontaneous imbibition, the surface charge of pore walls becomes important. We demonstrated that the electrostatic interactions between the elements in a rock–fluid system are the decisive factors during a nano-EOR process and any synergy between other EOR agents and nanomaterials should consider this factor. 

The inverse Bond number appears to be a very good approach for process understanding, although it only linked the additional recovery to IFT reduction in oil/brine systems without fully considering the wettability state. However, an analysis of the diffusion mechanism and some of the high recovery rates revealed that a substantial wettability alteration was taking place.

Some of the improvements presented here, especially in phase behavior, should be examined more closely. Better emulsification will not necessarily lead to higher recovery. It might also lead to clustering of the oil or low mobility of the emulsion phase. Similarly, on wettability state, drainage in a strongly water-wet medium is mainly achieved by oil-filling and snap-off, which might influence the relative permeability at pore scale. 

## Figures and Tables

**Table 1:** Overview of selected evaluations reported in the literature for nanoparticles or alkali.

Formulation	Approach	Rock Type	Takeaways	Ref.
NPs/Surfact.	IFT, Contact Angle, Rheology, Core Floods	Sandstones/Carbonates	NPs decreased IFT, wettability changes. Effects of NPs in IFT and wettability.	[2,3,5]
NPs/Surfact/Polymers	IFT, Contact Angle and Core Floods	Sandstones	Strong IFT reduction in brine/oil system when using nanoparticles	[9,16,17,24,25,26,28,30]
Alkali/Alkali Polymer	IFT, Core Floods, Phase Behavior	Sandstones	Favorable findings using alkali combined with polymer/surfactant	[36,37]
Alkali Polymer	IFT, Phase Behavior, Amott Imbibition	Sandstones	Alkali of IFT reduction in high-TAN oils with clear wettability changes.	[38]
Alkali Polymer	IFT, pH, Rheology	--	Alkali solution lowered IFT	[46]
Alkali Brine	IFT, NMR, Spontaneous Imbibition	Sandstones	Mixed impacts of alkali on the spontaneous imbibition kinetics	[47]
Alkali Polymer NPs	Unsteady State Displacement	Sandstones	Combination promoted favorable pressure gradient changes	[48]

**Table 2:** Composition of synthetic brines used in this work.

Formulation	TW Softened Injection Brine	FW Synthetic Formation Brine
NaCl [g/L]	18.96	19.75
NaHCO_3_ [g/L]	1.96	-
CaCl_2_ · 2 H_2_O [g/L]	-	0.40
MgCl_2_ · 6 H_2_O [g/L]	-	0.66
NH_4_Cl [g/L]	-	0.17
SrCl_2_ · 6 H_2_O [g/L]	-	0.06

**Table 3:** Composition of crude oils used in this work.

Property	High TAN	Low TAN
Reservoir/Well	16 TH/Bockfliess 112	St. Ulrich/St.U. 65
TVD top [m]	1622	1060
TAN [mg KOH/g]	1.61	0.39
Saturates [%]	39	55
Aromatics [%]	20	25.6
Resins [%]	39	18.6
Asphaltene [%]	2	0.8
Saponifiable Acids [µmol/g]	26	n.m.
µ @ 60 °C [mPa∙s]	11.9	6
ρ @ 20°C/60 °C [g/cm^3^]	0.917/0.884	0.866/0.842

n.m. = not measured.

**Table 4:** General properties of nanomaterials used in this work.

Property	Type A	Type B
Solid [wt. %]	22.5	27.9
m @ 10 1/s [mPa∙s]	16	39
Particle Size (d50)	110	114
pH	9.5	3.2

**Table 5:** Density and pH data for the fluids used in this work.

Fluid	Density ^1^ [g/cm^3^]		Ph ^2^ [-]	
	Mean	SD	Mean	SD
TW	0.994	0.01	8.93	0.01
FW	0.995	0.01	-	-
0.1 wt.% Type (A) in TW	0.997	0.01	8.56	0.02
0.1 wt.% Type (B) in TW	0.984	0.01	8.76	0.02
0.1 wt.% Type (A) + 3000 ppm Na_2_CO_3_ in TW	0.992	0.01	9.93	0.01
0.1 wt.% Type (B) + 3000 ppm Na_2_CO_3_ in TW	1.001	0.01	9.92	0.01
3000 ppm Na_2_CO_3_ in TW	0.998	0.01	9.98	0.01

^1^ Measured at 23 °C. ^2^ Measured at 22 °C.

**Table 6:** Overall core and saturation data for the outcrop samples used in this work.

Parameter	Units	Berea ^1^		Keuper ^2^	
		Mean	SD	Mean	SD
Length	cm	6.97	0.02	8.12	0.09
Diameter		2.96	0.01	2.98	0.01
Bulk Volume	cm^3^	47.76	0.26	55.76	0.73
Pore Volume		10.77	0.19	12.75	0.22
Grain Volume	kg/cm^3^	37.00	0.31	42.98	0.67
Porosity	%	22.60	0.40	23.30	0.80
*N*_2_ permeability (*k_g_*)	mD	447.60	37.40	1425.20	349.60
Water (Test Water) permeability (*k_w_*)		223.90	17.90	890.00	193.90
Irreducible water saturation	%	24.00	8.00	21.40	7.90

^1^ Data from 48 core plugs. ^2^ Data from 33 core plugs.

**Table 7:** Summary of IFT values of various oil/brine systems in this work.

Fluid	High TAN Oil [mN/m]		Low TAN Oil [mN/m]	
	Mean	SD	Mean	SD
Baseline (TW)	8.40	-	3.40	0.50
0.1 wt.% Nano A + TW	8.30	0.15	2.60	0.13
0.1 wt.% Nano B + TW	5.31	0.49	1.26	0.06
3000 ppm Na_2_CO_3_ + TW	0.11	0.01	0.55	0.15
0.1 wt.% Nano A + 3000 ppm Na_2_CO_3_ + TW	0.04	0.01	0.17	0.01
0.1 wt.% Nano B + 3000 ppm Na_2_CO_3_ + TW	0.06	0.01	1.00	0.01

**Table 8:** Summary of oil ultimate recoveries in Berea and Keuper core plugs using nanomaterials and alkali. Fluids were dissolved in TW for preparation.

Fluid	Berea Outcrop				Keuper Outcrop			
	High TAN Oil [%]		Low TAN Oil [%]		High TAN Oil [%]		Low TAN Oil [%]	
	Mean	SD	Mean	SD	Mean	SD	Mean	SD
Baseline (TW)	47.20	8.40	47.20	8.40	44.80	-	10.30	1.50
0.1 wt.% Nano A	67.10	0.15	67.10	0.15	41.10	2.20	13.80	3.40
0.1 wt.% Nano B	50.20	1.50	50.20	1.50	57.00	7.70	9.30	2.00
3000 ppm Na_2_CO_3_	59.70	23.10	59.70	23.10	95.30	2.60	41.00	15.10
0.1 wt.% Nano A + 3000 ppm Na_2_CO_3_	45.80	1.70	45.80	1.70	95.40	5.90	72.20	5.90
0.1 wt.% Nano B + 3000 ppm Na_2_CO_3_	97.70	16.01	97.70	16.01	97.50	19.01	66.80	22.70

**Figure A1:**
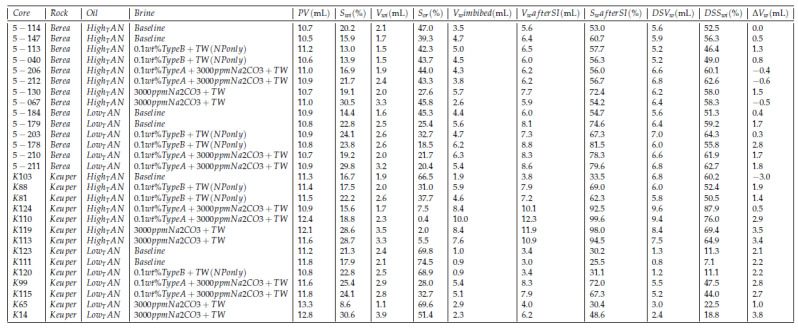
Dean–Stark data obtained in this work.

**Figure A2:**
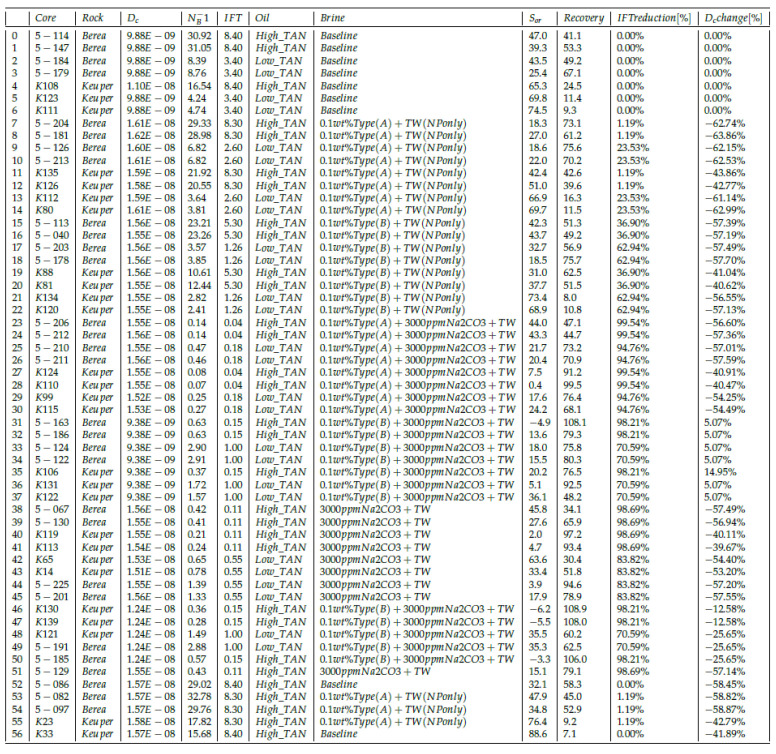
Calculations of inverse Bond number, NB^−1^ data obtained in this work.

**Figure A3:**
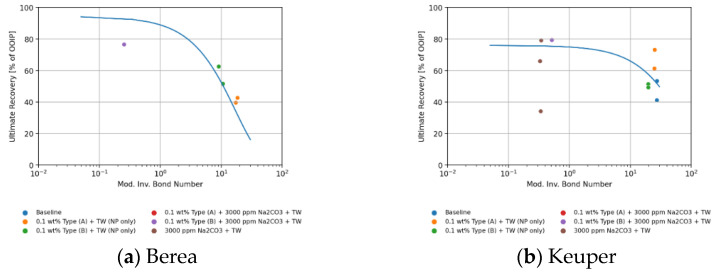
Ultimate recovery in % versus the modified inverse Bond number, NB^−1^ considering wettability (using Equation (8)) for the different cores (Berea and Keuper) for high TAN oil. No contact angle information is available for low TAN oil samples. While (**a**) shows the values obtained for selected Berea samples, (**b**) does for Keuper.

## Appendix A

Figure A1 summarizes the results of Dean–Stark extraction. By and large, the results were in line with the estimated recoveries. However, in core plugs, which were submerged in alkali and nanomaterials, the extracted water volumes were less than estimated, accompanied by high recoveries (exceeded 100% of initial oil saturation) in some cases. This can be explained by the spontaneous emulsification during the recovery process, which leads to overestimation of the recoveries since the readings corresponds for emulsion volume rather than pure oil volume.

## Appendix B

Figure A2 shows the additional data obtained for the calculations of the inverse Bond number, NB^−1^.

## References

[1] Jiang R., Li K., Horne R. A Mechanism Study of Wettability and Interfacial Tension for EOR Using Silica Nanoparticles. Proceedings of the SPE Annual Technical Conference and Exhibition.

[2] Joonaki E., Ghanaatian S. (2014). The Application of Nanofluids for Enhanced Oil Recovery: Effects on Interfacial Tension and Coreflooding Process. Pet. Sci. Technol..

[3] Sun Q., Li Z., Li S., Jiang L., Wang J., Wang P. (2014). Utilization of Surfactant-Stabilized Foam for Enhanced Oil Recovery by Adding Nanoparticles. Energy Fuels.

[4] Roustaei A., Saffarzadeh S., Mohammadi M. (2013). An evaluation of modified silica nanoparticles’ efficiency in enhancing oil recovery of light and intermediate oil reservoirs. Egypt. J. Pet..

[5] Kamal M.S., Adewunmi A.A., Sultan A.S., Al-Hamad M.F., Mehmood U. (2017). Recent Advances in Nanoparticles Enhanced Oil Recovery: Rheology, Interfacial Tension, Oil Recovery, and Wettability Alteration. J. Nanomater..

[6] Cheraghian G., Rostami S., Afrand M. (2020). Nanotechnology in Enhanced Oil Recovery. Processes.

[7] Rezk M.Y., Allam N.K. (2019). Impact of Nanotechnology on Enhanced Oil Recovery: A Mini-Review. Ind. Eng. Chem. Res..

[8] Kazemzadeh Y., Shojaei S., Riazi M., Sharifi M. (2019). Review on application of nanoparticles for EOR purposes: A critical review of the opportunities and challenges. Chin. J. Chem. Eng..

[9] Neubauer E., Hincapie R.E., Clemens T., Maximilian C. Selection of Nanomaterials as Emulsion Stabilizers in Alkali-Polymer EOR of High-TAN Number Oil. Proceedings of the SPE Improved Oil Recovery Conference.

[10] Neubauer E., Hincapie R.E., Borovina A., Biernat M., Clemens T., Ahmad Y.K. Influence of Nanofluids on Wettability Changes and Interfacial Tension Reduction. Proceedings of the SPE Europec.

[11] Raffa P., Druetta P. (2019). Chemical Enhanced Oil Recovery—Advances in Polymer Flooding and Nanotechnology.

[12] ShamsiJazeyi H., Miller C.A., Wong M.S., Tour J.M., Verduzco R. (2014). Polymer-Coated Nanoparticles for Enhanced Oil Recovery. J. Appl. Polym. Sci..

[13] Ali J.A., Kolo K., Manshad A.K., Mohammadi A.H. (2018). Recent advances in application of nanotechnology in chemical enhanced oil recovery: Effects of nanoparticles on wettability alteration, interfacial tension reduction, and flooding. Egypt. J. Pet..

[14] Singh R., Mohanty K.K. (2017). Foam flow in a layered, heterogeneous porous medium: A visualization study. Fuel.

[15] Corredor L.M., Aliabadian E., Husein M., Chen Z., Maini B., Sundararaj U. (2019). Heavy oil recovery by surface modified silica nanoparticle/HPAM nanofluids. Fuel.

[16] Sharma T., Kumar G.S., Chon B.H., Sangwai J.S. (2015). Thermal stability of oil-in-water Pickering emulsion in the presence of nanoparticle, surfactant, and polymer. J. Ind. Eng. Chem..

[17] Ahmed A., Saaid I.M., Ahmed A.A., Pilus R.M., Baig M.K. (2020). Evaluating the potential of surface-modified silica nanoparticles using internal olefin sulfonate for enhanced oil recovery. Pet. Sci..

[18] Kim I., Worthen A., Lotfollahi M., Johnston K., DiCarlo D., Chun Huh C. Nanoparticle-Stabilized Emulsions for Improved Mobility Control for Adverse-mobility Waterflooding. Proceedings of the SPE Improved Oil Recovery Conference.

[19] Arab D., Kantzas A., Bryant S. (2018). Nanoparticle stabilized oil in water emulsions: A critical review. J. Pet. Sci. Eng..

[20] Perazzo A., Tomaiuolo G., Preziosi V., Guido S. (2018). Emulsions in porous media: From single droplet behavior to applications for oil recovery. Adv. Colloid Interface Sci..

[21] Binks B., Rodrigues J.A. (2007). Enhanced Stabilization of Emulsions Due to Surfactant-Induced Nanoparticle Flocculation. Langmuir.

[22] Milad Kamkar M., Bazazi P., Kannan A., Chandran V.S., Hejazi S.H., Fuller G.G., Sundararaj U. (2020). Polymeric-nanofluids stabilized emulsions: Interfacial versus bulk rheology. J. Colloid Interface Sci..

[23] Juarez-Morejon J.L., Bertin H., Omari A., Hamon G., Cottin C., Bourdarot G., Morel D., EAGE (2017). Spontaneous Imbibition as Indicator of Wettability Change During Polymer Flooding. Proceedings of the IOR 2017—19th European Symposium on Improved Oil Recovery.

[24] Chengara A., Nikolov A.D., Wasan D.T., Trokhymchuk A., Henderson D. (2004). Spreading of nanofluids driven by the structural disjoining pressure gradient. J. Colloid Interface Sci..

[25] Wasan D., Nikolov A. (2003). Spreading of nanofluids on solids. Nature.

[26] Kuang W., Saraji S., Piri M. (2018). A systematic experimental investigation on the synergistic effects of aqueous nanofluids on interfacial properties and their implications for enhanced oil recovery. Fuel.

[27] Sofla S.J.D., James L.A., Zhang Y. (2019). Toward a mechanistic understanding of wettability alteration in reservoir rocks using silica nanoparticles. E3S Web Conf..

[28] Huh C., Daigle H., Prigiobbe V., Prodanović M. (2019). Practical Nanotechnology for Petroleum Engineers.

[29] Li S., Torsæter O., Lau H.C., Hadia N.J., Stubbs L.P. (2019). The Impact of Nanoparticle Adsorption on Transport and Wettability Alteration in Water-Wet Berea Sandstone: An Experimental Study. Front. Phys..

[30] Alvarez-Berrios M.P., Aponte-Reyes L.M., Aponte-Cruz L.M., Loman-Cortes P., Vivero-Escoto J.L. (2018). Effect of the surface charge of silica nanoparticles on oil recovery: Wettability alteration of sandstone cores and imbibition experiments. Int. Nano Lett..

[31] Bila A., Stensen J.Å., Torsæter O. (2019). Experimental Investigation of Polymer-Coated Silica Nanoparticles for Enhanced Oil Recovery. Nanomaterials.

[32] Li S., Dan D., Lau H.C., Hadia N.J., Torsæter O., Stubbs L.P. Investigation of Wettability Alteration by Silica Nanoparticles Through Advanced Surface-Wetting Visualization Techniques. Proceedings of the SPE Annual Technical Conference and Exhibition.

[33] Xu D., Bai B., Wu H., Hou J., Meng Z., Sun R., Li Z., Lu Y., Kang W. (2019). Mechanisms of imbibition enhanced oil recovery in low permeability reservoirs: Effect of IFT reduction and wettability alteration. Fuel.

[34] Wang X., Xiao S., Zhang Z., He J. (2017). Effect of Nanoparticles on Spontaneous Imbibition of Water into Ultraconfined Reservoir Capillary by Molecular Dynamics Simulation. Energies.

[35] Sheng J. (2015). Status of Alkaline Flooding Technology. J. Pet. Eng. Technol..

[36] Sheng J.J. (2016). Critical review of alkaline-polymer flooding. J. Pet. Explor. Prod. Technol..

[37] Sheng J.J. (2011). Modern Chemical Enhanced Oil Recovery.

[38] Arekhov V., Hincapie R.E., Clemens T., Tahir M. (2020). Variations in Wettability and Interfacial Tension during Alkali–Polymer Application for High and Low TAN Oils. Polymers.

[39] Schumi B., Clemens T., Wegner J., Ganzer L., Kaiser A., Leitenmüller L., Hincapie R. (2020). Alkali-Co-Solvent-Polymer Flooding of High TAN Number Oil: Using Phase Experiments, Micro-Models and Corefloods for Injection Agent Selection. SPE Res. Eval. Eng..

[40] Xu X., Saeedi A. (2017). Evaluation and Optimization Study on a Hybrid EOR Technique Named as Chemical-Alternating-Foam Floods. Oil Gas Sci. Technol.—Rev. IFP.

[41] Hu X., Li M., Peng C., Davarpanah A. (2020). Hybrid Thermal-Chemical Enhanced Oil Recovery Methods; An Experimental Study for Tight Reservoirs. Symmetry.

[42] Hamza M.F., Sinnathambi C.M., Merican Z.M. (2017). Recent advancement of hybrid materials used in chemical enhanced oil recovery (CEOR): A review. IOP Conf. Ser.: Mater. Sci. Eng..

[43] Druetta P., Picchioni F. (2020). Surfactant-Polymer Interactions in a Combined Enhanced Oil Recovery Flooding. Energies.

[44] Druetta P., Picchioni F. (2019). Polymer and nanoparticles flooding as a new method for Enhanced Oil Recovery. J. Pet. Sci. Eng..

[45] Rock A., Hincapie R.E., Hoffmann E., Ganzer L. Tertiary Low Salinity Waterflooding LSWF in Sandstone Reservoirs: Mechanisms, Synergies and Potentials in EOR Applications. Proceedings of the SPE Europec featured at 80th EAGE Conference and Exhibition.

[46] Maneeintr K., Meekoch T., Jongkittinarukorn K., Boonpramote T. (2020). Interfacial Tension Measurement for Alkaline-Polymer Flooding Application for Oil from Fang Oilfield, Thailand. Chem. Eng. Trans..

[47] Chevalier T., Labaume J., Delbos A., Clemens T., Waeger V.M., Bourbiaux B., Fleury M. (2019). A Practical Methodology to Screen Oil Recovery Processes Involving Spontaneous Imbibition. Transp. Porous Media.

[48] Mortazavi E., Masihi M., Ghazanfari M. (2016). An Influence of Polymer-Alkaline and Nanoparticles as Chemical Additives on the Immiscible Displacement and Phase Relative Permeability. Iran. J. Oil Gas Sci. Technol..

[49] French T., Burchfield T. Design and Optimization of Alkaline Flooding Formulations. Proceedings of the SPE/DOE Enhanced Oil Recovery Symposium.

[50] Lüftenegger M., Clemens T. Chromatography Effects in Alkali Surfactant Polymer Flooding. Proceedings of the SPE Europec Featured at 79th EAGE Conference and Exhibition.

[51] Bailey H.R., Gogarty W.B. (1963). Diffusion Coefficients from Capillary Flow. SPE J..

[52] Crank J. (1979). The Mathematics of Diffusion.

[53] Al-Quraishi A.A. (2004). Oil Recovery by Dynamic Imbibition in Low Tension Aqueous Systems. Oil Gas Sci. Technol.—Rev. IFP.

[54] Schechter D., Zhou D., Orr F.M. (1994). Low IFT drainage and imbibition. J. Pet. Sci. Eng..

[55] Babadagli T. (2005). Analysis of Oil Recovery by Spontaneous Imbibition of Surfactant Solution. Oil Gas Sci. Technol.—Rev. IFP.

[56] Chen H., Fan H., Zhang Y., Xu X., Liu L., Hou Q. (2018). Investigations on the driving forces of the fluorocarbon surfactant-assisted spontaneous imbibition using thermogravimetric analysis (TGA). RSC Adv..

[57] Vatanparast H., Shahabi F., Bahramian A., Javadi A., Miller R. (2018). The Role of Electrostatic Repulsion on Increasing Surface Activity of Anionic Surfactants in the Presence of Hydrophilic Silica Nanoparticles. Sci. Rep..

[58] Sharma M., Jang L., Yen T. (1989). Transient Interfacial Tension Behavior of Crude-Oil/Caustic Interfaces. SPE Reserv. Eng..

[59] Spanos N., Koutsoukos P.G. (1998). Kinetics of Precipitation of Calcium Carbonate in Alkaline pH at Constant Supersaturation. Spontaneous and Seeded Growth. J. Phys. Chem. B.

[60] Liesegang M., Milke R., Kranz C., Neusser G. (2017). Silica nanoparticle aggregation in calcite replacement reactions. Sci. Rep..

[61] Magnabosco G., Polishchuk I., Palomba F., Rampazzo E., Prodi L., Aizenberg J., Pokroy B., Falini G. (2019). Effect of Surface Chemistry on Incorporation of Nanoparticles within Calcite Single Crystals. Cryst. Growth Des..

[62] Morrow N.R., Mason G. (2001). Recovery of oil by spontaneous imbibition. Curr. Opin. Colloid Interface Sci..

[63] Zhou D., Jia L., Kamath J., Kovscek A.R. (2002). Scaling of counter-current imbibition processes in low-permeability porous media. J. Pet. Sci. Eng..

[64] Meng Q., Liu H., Wang J. (2017). A critical review on fundamental mechanisms of spontaneous imbibition and the impact of boundary condition, fluid viscosity and wettability. Adv. Geo-Energy Res..

[65] Sayyouh M., Dahab A., Omar A. (1990). Effect of clay content on wettability of sandstone reservoirs. J. Pet. Sci. Eng..

[66] Mohammed I., Al Shehri D., Mahmoud M., Kamal M.S., Alade O.S. (2021). Impact of Iron Minerals in Promoting Wettability Alterations in Reservoir Formations. ACS Omega.

[67] Omurlu C., Pham H., Nguyen Q.P. (2016). Interaction of surface-modified silica nanoparticles with clay minerals. Appl. Nanosci..

[68] Buckley J.S. (1995). Asphaltene Precipitation and Crude Oil Wetting. SPE Adv. Technol. Ser..

[69] Metin C.O., Lake L.W., Miranda C.R., Nguyen Q.P. (2011). Stability of aqueous silica nanoparticle dispersions. J. Nanopart. Res..

[70] Haagh M.E.J., Siretanu I., Duits M.H.G., Mugele F. (2017). Salinity-Dependent Contact Angle Alteration in oil/Brine/Silicate Systems: The Critical Role of Divalent Cations. Langmuir.

